# Antifoam addition to shake flask cultures of recombinant *Pichia pastoris *increases yield

**DOI:** 10.1186/1475-2859-10-17

**Published:** 2011-03-22

**Authors:** Sarah J Routledge, Christopher J Hewitt, Nagamani Bora, Roslyn M Bill

**Affiliations:** 1 School of Life and Health Sciences, Aston University, Aston Triangle, Birmingham B4 7ET, UK; 2 Centre for Biological Engineering, Department of Chemical Engineering, Loughborough University, Leicestershire LE11 3TU, UK

## Abstract

**Background:**

*Pichia pastoris *is a widely-used host for recombinant protein production. Initial screening for both suitable clones and optimum culture conditions is typically carried out in multi-well plates. This is followed by up-scaling either to shake-flasks or continuously stirred tank bioreactors. A particular problem in these formats is foaming, which is commonly prevented by the addition of chemical antifoaming agents. Intriguingly, antifoams are often added without prior consideration of their effect on the yeast cells, the protein product or the influence on downstream processes such as protein purification. In this study we characterised, for the first time, the effects of five commonly-used antifoaming agents on the total amount of recombinant green fluorescent protein (GFP) secreted from shake-flask cultures of this industrially-relevant yeast.

**Results:**

Addition of defined concentrations of Antifoam A (Sigma), Antifoam C (Sigma), J673A (Struktol), P2000 (Fluka) or SB2121 (Struktol) to shake-flask cultures of *P. pastoris *increased the total amount of recombinant GFP in the culture medium (the total yield) and in the case of P2000, SB2121 and J673A almost doubled it. When normalized to the culture density, the GFP specific yield (μg OD_595_^-1^) was only increased for Antifoam A, Antifoam C and J673A. Whilst none of the antifoams affected the growth rate of the cells, addition of P2000 or SB2121 was found to increase culture density. There was no correlation between total yield, specific yield or specific growth rate and the volumetric oxygen mass transfer coefficient (*k_L_a*) in the presence of antifoam. Moreover, the antifoams did not affect the dissolved oxygen concentration of the cultures. A comparison of the amount of GFP retained in the cell by flow cytometry with that in the culture medium by fluorimetry suggested that addition of Antifoam A, Antifoam C or J673A increased the specific yield of GFP by increasing the proportion secreted into the medium.

**Conclusions:**

We show that addition of a range of antifoaming agents to shake flask cultures of *P. pastoris *increases the total yield of the recombinant protein being produced. This is not only a simple method to increase the amount of protein in the culture, but our study also provides insight into how antifoams interact with microbial cell factories. Two mechanisms are apparent: one group of antifoams (Antifoam A, Antifoam C and J673A) increases the specific yield of GFP by increasing the total amount of protein produced and secreted per cell, whilst the second (P2000 or SB2121) increases the total yield by increasing the density of the culture.

## Background

The laboratory-scale production of recombinant proteins using *P. pastoris *requires that cells are cultured either in large shake flasks or in continuously stirred tank bioreactors. In these vessels, the formation of foam is an issue that requires intervention. This is in contrast to the situation in the small vessels typically used in the initial stages of protein production experiments where foaming is minimal [1].

Foaming can lead to reduced yields since bursting bubbles can damage proteins [2] and can also result in a loss of sterility if the foam escapes [3]. In bioreactors, foaming can lead to over-pressure if a foam-out blocks an exit filter. To prevent the formation of foam, mechanical foam breakers, ultrasound or, most often, the addition of chemical antifoaming agents (or "antifoams") [3] are routinely employed.

There is a well-established literature on antifoams [3]. One useful classification categorizes them as either hydrophobic solids dispersed in carrier oil, aqueous suspensions/emulsions, liquid single components or solids [4-6]. Several mechanisms of action for these agents have been suggested which include bridging-dewetting, spreading fluid entrainment and bridging-stretching [7]. Many are commercially-available, with 19 being sold by Sigma-Aldrich alone. While little information is routinely given about their composition, their specific antifoam properties have been thoroughly investigated. These include their effects on foam height with time, their influence on the volumetric oxygen mass transfer coefficient (*k*_L_a) of the system, their gas hold-up characteristics and their globule size and distribution in relation to their action upon foams [3,5,7-11]. Such studies have been performed in various growth media in both the absence and presence of cultures of prokaryotic and eukaryotic microbes.

In contrast, literature on the biological effects of antifoams on recombinant protein yields from microbial cell factories is more limited. Additional file 1: Table S1 shows an analysis of representative examples of this body of work including previous studies on four bacterial hosts and one yeast species. In some cases, the additives tested are not antifoams *sensu stricto*. It is also noteworthy that the yeast, *Schizosaccharomyces pombe*, is not widely used in biotechnology applications and that there have been no prior studies on the biological effects of antifoam addition to recombinant *P. pastoris *cultures. A recent review stated that in the last 15 years, 80% of all recombinant genes reported in the literature were expressed in either *Escherichia coli *or *P. pastoris *[12]. In this study, we therefore examined five antifoams that are widely used in controlling the foaming of recombinant *P. pastoris *cultures [13-16] in order to analyze effects over and above that of their de-foaming action. We looked at polypropylene glycol (PPG) P2000 that is analogous to previously-examined liquid single components of the PPG-type [11] as well as examples from other categories such as Antifoam A and Antifoam C, which are silicone polymers, SB2121, which is a polyalkylene glycol, and J673A, which is an alkoxylated fatty acid ester on a vegetable base and has not previously been documented in this context: for all antifoams examined, this was the first report of their effect on the yield of recombinant GFP secreted from shake-flask cultures of *P. pastoris*.

## Results

We wanted to establish whether antifoams affect recombinant protein yield in *P. pastoris *X33 cultures, and if so to investigate the underlying mechanisms. To examine this we chose an experimental system, under the control of the methanol-inducible *AOX1 *promoter, comprising GFP secreted from 20 mL cultures in shake flasks in the presence of five different antifoams at a range of concentrations from 0-1% v/v. These concentrations are higher than the 0.1% routinely used for de-foaming purposes. The total amount of GFP in these 20 mL cultures (the total yield) was measured by fluorimetry 48 h post-induction.

### Antifoam addition affects total GFP yield in shake flasks

**Figure 1:**
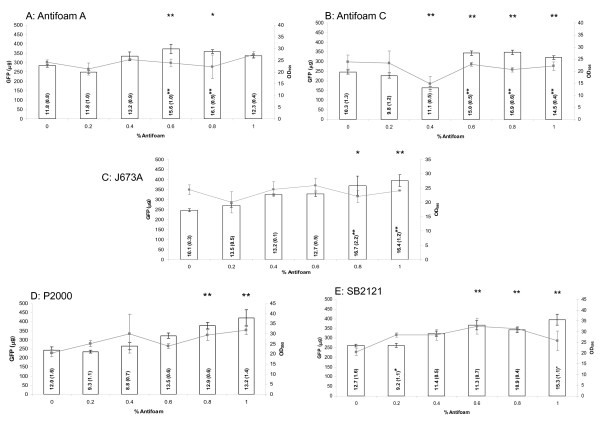
**Antifoam addition increases the total yield of GFP in 20 mL *P. pastoris *cultures**. Bar charts showing the total yield of GFP (μg) at 48 h in 20 mL *P. pastoris *cultures following addition of Antifoam A (A), Antifoam C (B), J673A (C), P2000 (D) and SB2121 (E) at concentrations from 0-1%. The error bars show the respective standard deviations. In all cases n = 9. The numbers within each bar are the corresponding specific yield (μg OD_595_^-1^) with the respective standard deviations in parentheses (n = 9). The horizontal line is a visual aid to link the mean optical density (grey squares) for each concentration of antifoam across the full experimental range; error bars show the respective standard deviations (n = 9). In each case a one-way ANOVA showed that P < 0.001. Asterisks show the significance of the total yield and specific yield data for each antifoam concentration compared to the respective 0% antifoam control as determined by a Dunnett's multiple comparison test, where * = P ≤ 0.05 and ** = P ≤ 0.01.

The total yield of GFP as a function of Antifoam A addition rose significantly at concentrations of 0.6% and above (Figure 1A) with no further increases above 1% (data not shown). A similar but more pronounced trend was observed for Antifoam C (Figure 1B), which is unsurprising since Antifoam C is a 30% emulsion of the same antifoam concentrate as Antifoam A, but with different non-ionic emulsifiers [17]. Figure 1C shows that addition of 1% J673A almost doubled the total yield of GFP compared to the control without antifoam, representing one of the largest effects of the antifoams evaluated. At concentrations above 1%, the total yield of GFP decreased (data not shown). Addition of P2000 (Figure 1D) also resulted in a significant increase in total yield at or above 0.6%, while addition of SB2121 (Figure 1E) increased total yield at concentrations above 0.4%. In both cases the largest improvement was obtained on addition of 1% of the antifoam, again almost doubling the yield. Overall, the five antifoams tested all increased the total yield of GFP at concentrations in the range of 0.4-1% v/v. The highest yield was achieved by adding 1% P2000 (422 μg GFP) followed by 1% SB2121 (396 μg GFP), 1% J673A (394 μg GFP), 0.6% Antifoam A (373 μg GFP) and 0.8% Antifoam C (348 μg GFP). All five yields were significantly higher than the corresponding yields from the 0% control, as shown in Figure 1.

### The effects of antifoam addition are due to changes in culture density for P2000 and SB2121

To account for any changes in the growth characteristics of the cells on addition of the antifoams, we normalized the total yield to the optical density of the cultures to obtain the specific yield (μg OD_595_^-1^). OD_595 _was demonstrated to be a reliable measure of cell density in these experiments by comparing the number of cells at a given OD_595 _in the absence and presence of a range of concentrations of the different antifoams used in our study: there was no statistically significant difference in cell number between cells harvested at a given OD_595 _in the absence or presence of all antifoam concentrations tested. Typical values were 4.8 × 10^7 ^cells/mL at an OD_595 _of 20.5 in the absence and presence of 0.5% SB2121.

For Antifoam A, Antifoam C and J673A, the specific yield data were similar in trend to the total yield data (Figure 1A-C): addition of these antifoams in the range 0.6-1% v/v caused a significant increase in specific yield compared to the control cultures with no antifoam. For cultures containing P2000 or SB2121, however, there was no statistically significant difference in the specific yield at each antifoam concentration compared with the control except for 1% SB2121 where P < 0.05 (Figure 1D-E). This suggested that the enhancements in total yield due to P2000 or SB2121 addition might be attributable to changed growth characteristics of the cells. The specific growth rates (μ) for cultures containing either 1% P2000 or 1% SB2121 were 0.17 h^-1 ^and 0.18 h^-1 ^respectively compared with 0.17 h^-1 ^for the control samples (0% antifoam) indicating that the growth characteristics during the log phase were not affected by the presence of the antifoams. However, we noted an increase in OD_595 _(at both 24 and 48 h) with increasing antifoam concentration for both antifoams (Figure 1D-E; 48 h data), which was less pronounced for Antifoam A, Antifoam C and J673A (Figure 1A-C). We concluded, therefore, that there was more than one mechanism of antifoam action: one due to changed culture density (P2000, SB2121) and a second due to increased cellular production levels of recombinant GFP (Antifoam A, Antifoam C, J673A).

### Antifoam addition does not affect cell viability

**Figure 2:**
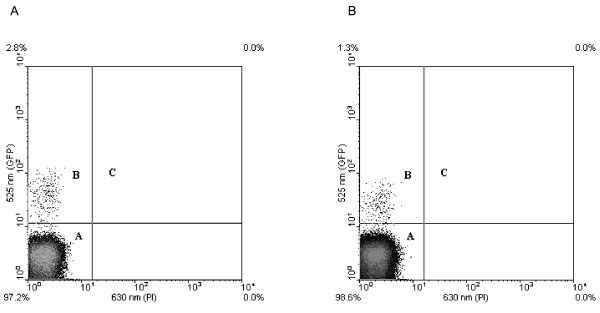
**Antifoams do not affect cell viability**. Viable cells without antifoam (A) and with 0.6% Antifoam A (as a representative example; B) are shown. Population A, which is not cellular, comprises events that are related to electronic and particulate noise. Population B comprises cells with enhanced green fluorescence due to the expression of GFP. Population C is where any dead cells (stained red with propidium iodide) would be observed.

We investigated the influence of antifoams on cell viability by propidium iodide exclusion and flow cytometry. In this assay, dead cells are stained red [18] and appear in population C (Figure 2) while live cells fluoresce green due to GFP production and appear in population B. The data shown in Figure 2A suggest that there are no dead cells present in cultures containing 0% antifoam. Figure 2B shows that the same result was obtained in the presence of 0.6% Antifoam A. This result was seen for all antifoams tested.

### The foam destruction capacity of an antifoam is related to its ability to improve GFP yield

**Figure 3:**
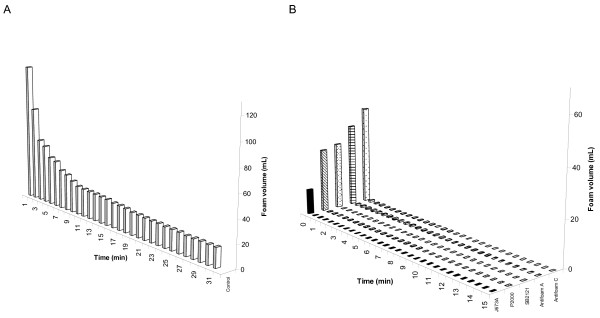
**Antifoams affect foam volume as determined by a Bartsch test**. The antifoams used in Figure 1, were tested for their foam destruction capacity. Foam volume was recorded for 0% v/v antifoam (A) and 0.001% of each antifoam (B) in BMMY medium over a 15 min time course (n = 5).

We wanted to understand how the five antifoams increase total yield and hence began by evaluating their foam destruction properties. Simple methods of determining the ability of antifoams to reduce foam are the Bartsch shaking test [19] and the Ross-Miles pouring test [20]. A Bartsch shaking test was conducted (Figure 3) and demonstrated that in the absence of an antifoaming agent, initial foam destruction was quick until a stable foam was formed. Foam height reduced slowly and in the 15 min testing time did not reach zero (Figure 3A). The most effective agent for foam reduction was J673A, where less foam was formed after initial shaking, and destruction was rapid. Antifoam C had the least activity of the agents tested. All antifoams were effective at foam destruction and most foam was destroyed within one minute (Figure 3B). Although there was no statistical correlation between foam destruction capacity and either total or specific yield, J673A was the most effective at foam destruction and one of the best at increasing GFP yield, whilst Antifoam C was the least effective.

### Improved yields cannot be explained by antifoam-induced changes in GFP fluorescence

In order to determine whether any of the antifoams affected the fluorescence of GFP, 1% antifoam was incubated in BMMY for 48 h to mimic the experimental set-up. This was then spiked with a similar concentration of recombinant GFP standard to that obtained in Figure 1. There was no significant difference between the fluorescence of GFP in the presence and absence of any of the antifoams suggesting that they did not influence the sample readings. The fluorescence values of the antifoams themselves were also measured at 1% and found to be minimal, similar to the buffer control readings.

### The *k_L_a *characteristics of antifoam-containing cultures are not correlated with improvements in GFP yield

**Figure 4:**
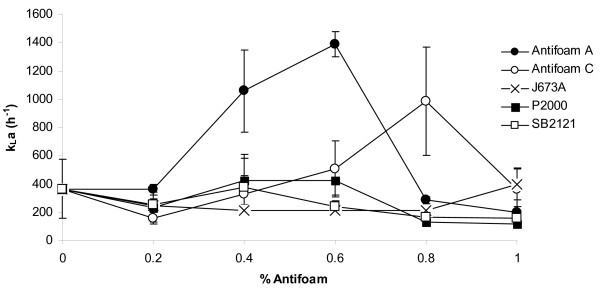
***k*_L_a data for the antifoam panel**. The antifoams used in Figure 1, were tested to examine how their addition affected *k*_L_a. Values were recorded for 0-1% v/v antifoam for Antifoam A (closed circles), Antifoam C (open circles), J673A (crosses), P2000 (closed squares) and SB2121 (open squares).

As P2000 and SB2121 affected the density of the cultures, we investigated the possibility that the oxygen availability in the system was affected by antifoam addition and that this could explain increased GFP yields. The *k_L_a *was therefore measured in shake flasks in the presence of 0-1% v/v of these antifoams. Addition of 0.4% or 0.6% Antifoam A caused a large increase in *k_L_a *compared to the control (Figure 4A), whereas addition of the related antifoam, Antifoam C, led to an initial reduction in *k_L_a*, which increased on addition of antifoam up to 0.8% and then returned to control levels at 1%. After an initial decrease in *k_L_a *was caused by J673A addition up to 0.4%, it remained relatively constant up to 1%. Addition of P2000 at all concentrations tested caused relatively minor changes to the *k_L_a*. SB2121 addition did not substantially increase the *k_L_a *at any of the concentrations tested. Overall, there was no correlation between *k_L_a *and total yield for any of the conditions tested, or with the density of the cultures, suggesting that changes in *k_L_a *may not directly lead to increased protein yield for these antifoams.

### DO in shake flasks is not affected by the presence of antifoams P2000 and SB2121

**Figure 5:**
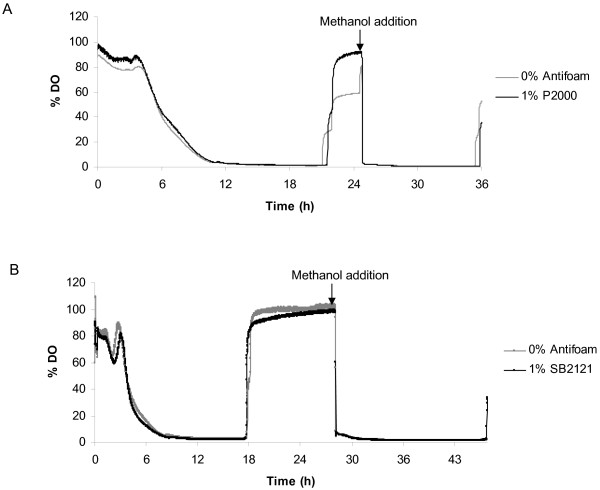
**P2000 and SB2121 do not affect DO in shake flasks**. Addition of 1% P2000 (A) or 1% SB2121 (B) did not affect the DO in shake flasks, as determined by DO fluorescent sensors (PreSens) attached to the underside of each flask. The experiments in panels A and B are biologically independent, which is the origin of the clonal differences and the variations in technical additions.

In addition to measuring *k_L_a*, we looked at the dissolved oxygen (DO) content of the cultures. The effect of 1% P2000 or 1% SB2121 addition on DO was assessed in shake flask cultures with PreSens DO patches and online monitoring. Figure 5 shows that there was no difference in DO in the flasks in the presence or absence of antifoam: after approximately 12 h for each culture condition the DO in the flasks became limiting. Since functional GFP can be expressed by anaerobic bacteria and in media containing 0.1 ppm dissolved oxygen [21], there was no concern that this would influence our data. DO decreased as the cells metabolized the methanol present in the medium and rose once they had consumed it. DO remained high until additional methanol was added at which point the DO immediately decreased and utilization continued. Methanol concentrations were confirmed by gas chromatographic analysis (data not shown). Overall, there was no difference in the DO content of cultures containing antifoam and those without.

### Addition of Antifoam A, Antifoam C or J637A affects the total yield of GFP secreted into the medium

**Figure 6:**
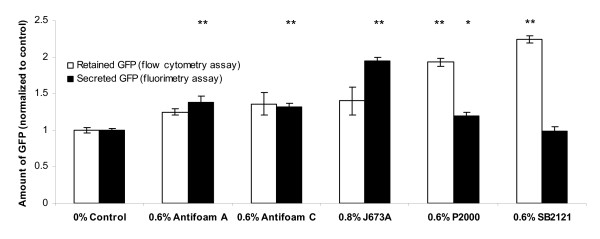
**Antifoam addition can affect both retained and secreted GFP**. The amount of GFP retained in the cell was analyzed by flow cytometry (white bars), while that secreted into the culture medium was analyzed by fluorimetry (black bars). In order to compare these two different data sets, each was normalized to its respective 0% antifoam control, which was consequently set to 1. The values shown are for determinations at 48 h (n = 3). The significance of the changes in retained (white bars) and/or secreted (black bars) GFP compared to the respective 0% antifoam control was analyzed by a one-way ANOVA (P < 0.0001) and a Dunnett's multiple comparison test where * = P ≤ 0.05 and ** = P ≤ 0.01.

We next investigated whether antifoam addition might have a physical influence on the cells. We therefore measured the amount of GFP retained in the cell (by flow cytometry) and that in the culture medium (by fluorimetry). Figure 6 shows that addition of Antifoam A, Antifoam C and J673A caused a statistically significant increase (P < 0.01) in the amount of GFP secreted into the medium compared with the 0% antifoam control. The amount of protein retained in the cells was also greater suggesting that antifoam addition enhanced the ability of the cells to produce recombinant GFP. For P2000 however, more GFP was retained inside the cells compared with the 0% antifoam control. This is consistent with the growth of the cells being affected by P2000 addition rather than resulting in improved secretion efficiency, and also suggests that there has been some metabolic change to the cells compared to the control. Data for SB2121 was similar to that for P2000. We also noted that addition of antifoam did not cause any change in the total concentration of all proteins in the supernatant (measured using a bicinchoninic acid (BCA) assay) for cultures containing antifoams at representative concentrations of 0%, 0.5% and 1%, except for 0.5% Antifoam C (P < 0.05) and 1% SB2121 ( P < 0.01). In the presence of these 2 antifoam concentrations, a decrease of 13-14% was observed in the total protein concentration of the supernatant compared to 0% antifoam-containing control cultures.

## Discussion

Antifoams have previously been suggested to alter the growth of cells and influence protein yield in bioprocesses [2,11], but their addition to *P. pastoris *cultures has never been examined systematically. In this study, addition of the five antifoams tested increased the total yield of secreted recombinant GFP produced by 20 mL *P. pastoris *cultures. Generally, the total GFP yield secreted into the culture medium was increased when antifoam was added at concentrations of at least 0.4% v/v compared with the 0% antifoam control. Addition of 1% J673A to a 20 mL culture yielded 394 μg GFP compared with 246 μg GFP when there was no antifoam present. This is notable as J673A is approved for industrial use.

Antifoams can be split into two categories of fast and slow antifoams, depending on their mechanism of foam destruction. Slow antifoams are often oils which destroy foam over a longer period of time. Fast antifoams, as examined in this study, are generally mixed agents which enter the foam film and destroy it by a bridging-stretching mechanism [7]. It has also been observed that the most effective agents at destroying foam are those with the most efficient oil film spreading characteristics [22]. The least effective de-foaming agents in this study were Antifoams A and C, which are 30% aqueous emulsions of Antifoam A concentrate [17]. Their reduced de-foaming capability was accompanied by the weakest ability to increase the yield of protein. In contrast, J673A addition produced one of the best results, almost doubling the yield. This antifoam is an alkoxylated fatty acid ester on a vegetable oil base and it is known that vegetable oils can alter the structure of foams by increasing bubble size and reducing the stability of the foam [23]. While vegetable oils may be metabolized as a carbon source [23], which might explain why J673A addition enhanced the yield of GFP, our data show that J673A did not influence the growth of the cells (Figure 1C), but rather enhanced the amount secreted into the medium (Figure 6). J673A was additionally found to be the most effective de-foamer of the panel of five antifoams that we assayed (Figure 3).

Antifoams are also known to affect the *k_L_a *of a system, which can be influenced by several factors such as medium viscosity, the presence of organisms and their by-products. These variables affect both *k_L _*(ms^-1^) and *a *(specific surface area m^-1^) [24,25]. For example, antifoam addition is known to have an effect [26] by enhancing bubble coalescence and increasing bubble size which leads to a reduction in the specific surface area thereby lowering *k_L_a *[3,8,24,25,27]. However, it has also been previously observed [25,28] that at higher concentrations of antifoam the *k_L_a *rises possibly due to the detrimental effects of bubble coalescence. Consequently the reduced specific surface area (*a*) reaches a limit and bubbles coalesce suppressing surface motility and decreasing surface tension. This then leads to decreasing bubble size and *k_L_a *rises again. Additionally it is possible that antifoams accumulate oxygen from rising bubbles, as they have good oxygen solubility, and then release it to the aqueous phase. Bubbles bursting at the surface disperse small drops of antifoam causing more oxygen to be released [25,28]. In the case of oils which have a greater oxygen solubility than water, oil droplets may increase oxygen permeability in the water boundary layer of the gaseous dispersion [23]. Yagi and colleagues suggested that surfactants can cause rippling or eddying which influences the *k_L_a *[26]. They also found that *k_L _*was not greatly affected by antifoams, but that their main effect was on *a *[26]. Koide subsequently suggested that the ability of antifoams to reduce *k_L _*is less for bubble swarms than for a single bubble [29].

We found that in shake flasks, the *k_L_a *was higher at mid-range values and decreased with increasing concentration, but that there was no statistically significant correlation between increased *k_L_a *and total yield. It is therefore possible that the *k_L_a *is already sufficient for the cells to grow and produce protein and is not a limiting factor, or that a combination of factors is responsible for the increases in total yield that we observed. This is supported by the DO shake flask data which suggest there is no difference between the DO in flasks without antifoam and those with either P2000 or SB2121 added. Combining flow cytometry and fluorimetry data showed that the antifoams can influence the amount of GFP retained inside the cell as well as the amount secreted into the medium. Antifoam A, Antifoam C and J673A enhanced the GFP secreted compared to 0% antifoam suggesting that the increase in total yield observed could be due to this secretion effect. This is consistent with an earlier study which suggested that antifoams can affect cell permeability in yeast by perturbing sterol biosynthesis which then alters the permeability of the membrane [30]. This is currently under investigation.

## Conclusions

We show that when Antifoam A, Antifoam C, J673A, P2000 or SB2121 are added at concentrations higher than those routinely used for de-foaming purposes, they all increase the total yield of shake-flask cultures. Two effects are apparent: one group of antifoams (Antifoam A, Antifoam C and J673A) increases the specific yield of GFP by increasing the total amount of protein produced and secreted per cell, whilst the second (P2000 or SB2121) increases the total yield by increasing the density of the culture. Addition of commonly-used antifoaming agents to shake flask cultures of yeast is therefore an effective way to increase the total yield of the recombinant protein being produced; any necessary changes to downstream steps such as protein purification are therefore likely to be worthwhile. Furthermore, this study provides insight into the manner in which antifoams interact with microbial cell factories: any data contributing to a fuller understanding of the specific effects of an antifoam on the growth and yield characteristics of such cultures, in addition to its primary action as a de-foamer, will be essential in bioprocess optimisation. These findings should provide an impetus to increase productivity in shake flask cultures of *P. pastoris*.

## Methods

### Yeast strains and culturing conditions

*Pichia pastoris *strain X33 transformed with pPICZαA-GFPuv (designated X33GFPuv) [1] was used in all experimental procedures. Cells were cultured in shake flasks in medium buffered to pH 6.0 with 1 M potassium phosphate buffer and at 30 °C and 220 rpm. BMGY medium (1% yeast extract, 2% peptone, 100 mM potassium phosphate pH 6.0, 1.34% YNB, 4 × 10^-5^% biotin, 1% glycerol [31]) was used for the initial biomass accumulation stage before transferring to the induction medium, BMMY (1% yeast extract, 2% peptone, 100 mM potassium phosphate pH 6.0, 1.34% YNB, 4 × 10^-5^% biotin, 0.5% methanol [31]), to induce production of GFP.

### Shake flask cultures

Cells were cultured in 50 mL BMGY in 250 mL baffled shake flasks to accumulate biomass. 20 mL BMMY was then inoculated to a final OD_595 _of 1.0 and transferred to a 100 mL non-baffled shake flasks for antifoam evaluations. Each evaluation was done in triplicate, with each flask containing the desired concentration of antifoam (0%, 0.2%, 0.4%, 0.6%, 0.8% or 1.0% (v/v)) with incubation at 30 °C and 220 rpm. After 24 h, 100% sterile methanol was added to 1% v/v to maintain production of GFP [1]. All optical density measurements were blanked against the relevant antifoam-containing medium. Since the antifoams themselves might influence OD_595_, we analyzed the relationship between OD_595 _readings in the absence and presence of a range of concentrations of different antifoams. In all cases the pair wise relationship was linear (R^2 ^was 0.91-0.99). We further verified that OD_595 _was a reliable measure of cell density by comparing the number of cells at a given OD_595 _in the absence and presence of a range of concentrations of different antifoams. There was no statistically significant difference in cell number between cells harvested at a given OD_595 _in the absence or presence of any of these antifoam concentrations, suggesting that OD_595 _is indeed a robust measurement of cell density.

### Antifoam agents

The antifoams tested in this study were Schill and Schelinger's Struktol SB2121 (a polyalkylene glycol), Schill and Schelinger's Struktol J673A (an alkoxylated fatty acid ester on a vegetable base), Fluka P2000 (a polypropylene glycol), Sigma Antifoam A (a 30% emulsion of silicone polymer) and Sigma Antifoam C (a 30% emulsion of silicone polymer). All antifoams were autoclaved prior to use and each shake flask experiment was performed in triplicate, with the undiluted antifoam being added directly to the medium.

### Fluorescence measurements

Culture supernatants (100 μL) were assayed at 24 h and 48 h post-induction for GFP fluorescence using a Spectramax Gemini XS plate reader with an excitation wavelength (λ_exe_) of 397 nm, and emission wavelength (λ_em_) of 506 nm. Triplicate determinations were performed for each independent sample. All samples and blanks were buffered to pH >7.0 using 50 μL 1 M potassium phosphate pH 7.5. Data were collected at 25 °C. To determine the concentration of GFP in each of the samples, a recombinant GFP standard (Vector Laboratories Ltd) was used to construct a standard curve relating RFU to protein concentration, as previously described [1]. All data were analyzed using a one-way ANOVA to test for a significant difference between any of the means. In all cases P < 0.001 indicating a high degree of significance. A Dunnett's multiple comparison test was then performed to compare each treatment mean (addition of various antifoam concentrations) and the control mean (0% antifoam).

### Total protein analysis

The total protein content of culture supernatants (2 μl) at 48 h post-induction was analyzed by bicinchoninic acid (BCA) assay. Cultures were examined in the presence of representative concentrations of 0%, 0.5% and 1% antifoam. 4.9 mL of proprietary BCA solution (B9643, Sigma) was mixed with 100 μL 4% mM copper (II) sulfate solution (C2284, Sigma). 200 μL of this solution was used to assay each independent supernatant sample in duplicate using a plate reader (BioTek Instruments) at 570 nm. To determine the concentration of protein in the samples, a bovine serum albumin standard (Sigma) was used to plot a standard curve. The data were analyzed using a one-way ANOVA (P < 0.0001) and a Dunnett's multiple comparison test.

### Bartsch antifoam test

Bartsch tests were conducted following a protocol adapted from that outlined by Denkov and colleagues [32]. A 500 mL graduated glass cylinder was filled with 166 mL BMMY medium and in all cases except for the control, antifoam was added to 0.01% v/v. The cylinder was sealed with parafilm and shaken ten times at ambient temperature. The height of the foam was recorded using the graduations on the cylinder every 30 s for 15 min. Determinations were performed in quintuplet for each antifoam. The activity of a given antifoam was reported as a volume [32], obtained by subtracting the volume of medium from the total volume (foam plus medium) in the cylinder.

### *k_L_a *determination

The influence of each antifoam on the volumetric mass oxygen transfer coefficient (*k_L_a*) in 125 mL plastic non-baffled shake flasks with DO fluorescent sensors (PreSens; the closest available size to our previous experimental set-up using the same total:working volume ratio of 5:1) was measured using a dynamic method adapted from that of Bandyopadhyay and Humphrey [33]. A working volume of 25 mL BMMY was used for each determination, with each antifoam being added in a stepwise manner to a final concentration of 0%, 0.2%, 0.4%, 0.6%, 0.8% and finally 1.0% (v/v). Shake flasks were sealed with foam bungs and incubated at 220 rpm, 30°C. The medium was saturated with 1.5 L min^-1 ^compressed air and flushed with N_2_. Determination of the *k_L_a *was carried out in triplicate by adding the required volume of antifoam at 100% DO, flushing with N_2 _until the DO was 0% and then allowing the DO to return to 100%. The data were logged every second using SFR software (PreSens). The data logged during the increase in DO from 0% to 100% were used to calculate the *k_L_a *with the following formula, where t_1 _and t_2 _are consecutive time points, c_1,t1 _is the oxygen concentration at time t_1 _and c_1,∞ _is the oxygen saturation concentration.

### Dissolved oxygen measurements

Dissolved oxygen was measured in 125 mL non-baffled shake flasks with DO fluorescent sensors (PreSens) attached to the underside of each flask. The flasks were placed on a shake flask reader which excites the dyes in the sensors and allows the DO data to be logged over 48 h with SFR software (Presens).

### Flow cytometry

Shake flask cultures of *P. pastoris*, as described above, were used to generate samples for flow cytometry analysis. The antifoams used were Antifoam A at 0.6%, Antifoam C at 0.6%, J673A at 0.8%, P2000 at 0.6% and SB2121 at 0.6% (v/v). Triplicate flasks were used for each antifoam. 48 h samples were diluted 1:1000 in phosphate buffered saline to a final concentration of 10^6^-10^7 ^cells mL^-1^, as determined using a haemocytometer. Fluorescent measurements were made using a Beckman Coulter (High Wycombe, UK) flow cytometer with λ_exe _= 488 nm from an argon-ion laser at 15 mW. Diluted samples were additionally stained with 10 μL propidium iodide (PI; 1 mgmL^-1 ^in water). All solutions were passed through a 0.2 μm filter, immediately prior to use, to remove particulate contamination. The optical filters were set up so that PI fluorescence was measured at 630 nm and GFP fluorescence was measured at 525 nm. The data were analyzed using a one-way ANOVA (P < 0.0001) and a Dunnett's multiple comparison test.

## Competing interests

The authors declare that they have no competing interests.

## Authors' contributions

SR was involved in all aspects of the experimental design, data collection, data analysis and interpretation and was supported by NB. CH oversaw the experimental design and data analysis for the flow cytometry component of the study. RB directed the study, coordinated the data analysis and interpretation and, together with SR, drafted the manuscript. All authors contributed to, read and approved the final version of the manuscript.

## Supplementary Material

Additional file 1 Table S1: Summary of the biological effects of antifoam addition to microbial cell factories Click here for file
