# Supplementary material for: Antifoam addition to shake flask cultures of recombinant Pichia pastoris increases yield
Source: Microb Cell Fact. 2011 Mar 22;10:17. doi: 10.1186/1475-2859-10-17 (PMC3072307; doi:10.1186/1475-2859-10-17)
Supplement: Additional file 1 — Table S1: Summary of the biological effects of antifoam addition to microbial cell factories [file 1475-2859-10-17-S1.DOC]

## Table S1 – Summary of the biological effects of antifoam addition to microbial cell factories

| **Microbial cell factory** | **Vessel** | **Antifoam**  **(category; composition)** | **Marketed as an antifoam?** | **Effect on recombinant protein yield?** | **Effect on growth rate of cells?** | **Other observations** | **Reference** |
| --- | --- | --- | --- | --- | --- | --- | --- |
| Prokaryotes |  |  |  |  |  |  |  |
| *Escherichia coli* K-12 producing β-galactosidase fusion protein | Bioreactor  (2 L and 60 L working volume) | S184  (Liquid single component; silicon oil) | No – has little effect on foaming | Reduces specific activity (mUg^-1^ dry cell mass) | No change below 250 ppm  No data reported above 250 ppm | OTR reduced in early stages of cultivation | [11] |
|  |  | SLM54474  (Liquid single component; polypropylene glycol) | Yes | Reduces specific activity | Decreases with increasing concentration | Minimal effect on OTR and *k*_L_a | [11] |
|  |  | VP1133  (Liquid single component; silicon oil/ polypropylene glycol mixture) | Yes | No effect | No change below 250 ppm  No data reported above 250 ppm | OTR reduced in early stages of cultivation | [11] |
|  |  | SE9  Aqueous emulsion of S184 silicon oil) | Yes | Increases volumetric activity (mU) | Low μ at 555 ppm  High μ at 5000 ppm | OTR reduced in early stages of cultivation | [11] |
| *Escherichia coli* K-12 producing benzaldehydelyase | Bioreactor  (3 L working volume) | Fluorocarbon-hydrocarbon hybrid unsymmetrical bolaform surfactant: FHUB  (Liquid single component; FHUB) | Yes | No effect | No effect | As effective an antifoam as Antifoam A | [9] |
| *Geobacillus thermoleovorans* secreting α-amylase | Shake flasks | PEG8000  (Liquid single component; polyethylene glycol) | Yes | Increases α-amylase titre (U mL^-1^ culture medium) at  0.5 % | No effect | Increased membrane permeability hypothesized | [34] |
|  |  | Tween-20  (Liquid single component; polyoxyethylene (20) sorbitan monolaurate) | No, more widely used as a detergent | Increases α-amylase titre at 0.3 % | No effect | As above | [34] |
|  |  | Tween-40  (Liquid single component; polyoxyethylene (40) sorbitan monolaurate) | No, more widely used as a detergent | Increases α-amylase titre at 0.3 % | No effect | As above | [34] |
|  |  | Tween-60  (Liquid single component; polyoxyethylene (60) sorbitan monolaurate) | No, more widely used as a detergent | Increases α-amylase titre at 0.3 % | No effect | As above | [34] |
|  |  | SDS, Tween-80 and cholic acid mixture  (Liquid single component; sodium dodecyl sulfate, polyoxyethylene (80) sorbitan monolaurate and cholic acid) | No | Increases α-amylase titre by a factor of 2 | No effect | As above | [34] |
|  |  | TritonX-100  (Liquid single component; polyethylene glycol p-(1,1,3,3-tetramethylbutyl)-phenyl ether) | No, more widely used as a detergent | Reduces Increases α-amylase titre | Not reported | None | [34] |
| *Bacillus subtilis* secreting α-amylase | Bioreactor | PEG600  (Liquid single component; polyethylene glycol) | Yes | Increases productivity (U mL^-1^ h^-1^) by a factor of 1.5 at 20 % | Not reported | Cells become “more hydrophilic” as measured by aqueous two-phase partition | [35] |
| *Bacillus amyloliquefaciens* secreting α-amylase | Bioreactor | PEG600  (Liquid single component; polyethylene glycol) | Yes | Reduces productivity by a factor of 2 at  20 % | Not reported | Cells become “more hydrophobic” as measured by aqueous two-phase partition | [35] |
| Eukaryotes |  |  |  |  |  |  |  |
| *Schizosaccharomyces pombe* secreting human transferrin | Not clearly specified; probably shake flasks | Dextran sodium sulphate; 0.001 – 0.1 %  (solid) | No | Increases titre (relative Western blot signal from 500 μL culture supernatant) by a factor of 7 | No effect | Anionic residues hypothesized to be important for exocytosis | [36] |
|  |  | SDS; 0.001 – 0.1 %  (Liquid single component; sodium dodecyl sulfate) | No, more widely used as a detergent | Not reported | Cells do not grow at > 0.001 % | None | [36] |
|  |  | Deoxycholate  (solid) | No | Increases titre at concentrations < 0.01 % | Not reported | None | [36] |
|  |  | Tween-20  (Liquid single component; polyoxyethylene (20) sorbitan monolaurate) | No, more widely used as a detergent | Decreases titre | Growth defect at > 0.001 % | None | [36] |
|  |  | TritonX-100; 0.002 %  (Liquid single component; polyethylene glycol p-(1,1,3,3-tetramethylbutyl)-phenyl ether) | No, more widely used as a detergent | No effect | No effect | None | [36] |
|  |  | PEG8000  (Liquid single component; polyethylene glycol) | Yes | Increases titre at 0.1 % | Growth defect at 1 % | None | [36] |
| *Pichia pastoris* secreting green fluorescent protein (GFP) | 100 mL shake flask containing 20 mL culture | Sigma Antifoam A  (Aqueous emulsion; 30% emulsion of silicone polymer) | Yes | 0.6 % – 1% increases total yield (total amount of GFP in 20 mL culture 48 h post-induction) | No | 0.6 % increases secretion and retention of GFP | This study |
|  |  | Sigma Antifoam C  (Aqueous emulsion; 30% emulsion of silicone polymer) | Yes | 0.6 % – 1 % increases total yield | No | 0.6 % increases secretion and retention of GFP | This study |
|  |  | Struktol J673A  (Hydrophobic solid dispersed in carrier oil; alkoxylated fatty acid ester on a vegetable base) | Yes | 0.4 % to 1 % increases total yield; 0.8 % doubles total yield | No | 0.8 % increases proportion of GFP secreted and doubles secretion compared to control | This study |
|  |  | Fluka P2000  (Liquid single component; polypropylene glycol) | Yes | 0.6 % to 1 % increases total yield; 1 % doubles total yield | No effect | 0.6 % increases proportion of GFP retained | This study |
|  |  | Struktol SB2121  (Liquid single component; polyalkylene glycol) |  | 0.4 % to 1 % increases total yield; 1 % doubles total yield | No effect | 0.6 % increases proportion of GFP retained | This study |

Data from the references cited were analysed for details of the experimental set-up (vessel, concentration of antifoam used) and whether the addition of the given antifoam (or other additive as part of the same study) affected the yield or growth characteristics of the cells. The yield data are reported according to the units specified in the respective reference. The data for recombinant *P. pastoris* cultures were generated as part of this study.
